# Increasing Antimicrobial Resistance in Surgical Wards at Mulago National Referral Hospital, Uganda, from 2014 to 2018—Cause for Concern?

**DOI:** 10.3390/tropicalmed6020082

**Published:** 2021-05-19

**Authors:** Gerald Mboowa, Dickson Aruhomukama, Ivan Sserwadda, Freddy Eric Kitutu, Hayk Davtyan, Philip Owiti, Edward Mberu Kamau, Wendemagegn Enbiale, Anthony Reid, Douglas Bulafu, Jeffrey Kisukye, Margaret Lubwama, Henry Kajumbula

**Affiliations:** 1 The African Center of Excellence in Bioinformatics and Data-Intensive Sciences, The Infectious Diseases Institute, Makerere University, Kampala P.O. Box 22418, Uganda; 2 Department of Immunology and Molecular Biology, College of Health Sciences, Makerere University, Kampala P.O. Box 7072, Uganda; dickson.aruhomukama@chs.mak.ac.ug (D.A.); ivangunz23@gmail.com (I.S.); 3 Department of Medical Microbiology, College of Health Sciences, Makerere University, Kampala P.O. Box 7072, Uganda; jkis8751@gmail.com (J.K.); maggienalum@gmail.com (M.L.); henrykajumbula427@gmail.com (H.K.); 4 Strengthening Pharmaceutical Systems (SPS) Unit, Pharmacy Department, School of Health Sciences, Makerere University, Kampala P.O. Box 7072, Uganda; kitutufred@gmail.com; 5 Tuberculosis Research and Prevention Center NGO, Yerevan 0014, Armenia; haykdav@gmail.com; 6 Academic Model Providing Access to Healthcare (AMPATH), P.O. Box 9505, Eldoret 30100, Kenya; philip.owiti@gmail.com; 7 Research Capacity Strengthening, Special Programme for Research and Training in Tropical Diseases, 1201 Geneva, Switzerland; kamaued@who.int; 8 Department of Dermatovenerology, College of Medicine and Health Sciences, Bahir Dar University, Bahir Dar P.O. Box 1996, Ethiopia; wendaab@gmail.com; 9 Amsterdam UMC, Academic Medical Centre, Department of Dermatology, Amsterdam Institute for Infection and Immunity (AI&I), University of Amsterdam, 1012 Amsterdam, The Netherlands; 10 Médecins Sans Frontières, Operational Centre Brussels, Operational Research Unit, 1617 Luxembourg, Luxembourg; Tony.Reid@brussels.msf.org; 11 Department of Environmental Health Sciences, College of Health Sciences, Makerere University, Kampala P.O. Box 7072, Uganda; bulafudouglas@gmail.com

**Keywords:** antimicrobial resistance (AMR), trends, Structured Operational Research and Training IniTiative (SORT IT), surgical wards, Mulago National Referral Hospital (MNRH), Uganda

## Abstract

Antimicrobial Resistance (AMR) and Healthcare Associated Infections (HAIs) are major global public health challenges in our time. This study provides a broader and updated overview of AMR trends in surgical wards of Mulago National Referral Hospital (MNRH) between 2014 and 2018. Laboratory data on the antimicrobial susceptibility profiles of bacterial isolates from 428 patient samples were available. The most common samples were as follows: tracheal aspirates (36.5%), pus swabs (28.0%), and blood (20.6%). Klebsiella (21.7%), Acinetobacter (17.5%), and Staphylococcus species (12.4%) were the most common isolates. The resistance patterns for different antimicrobials were: penicillins (40–100%), cephalosporins (30–100%), *β*-lactamase inhibitor combinations (70–100%), carbapenems (10–100%), polymyxin E (0–7%), aminoglycosides (50–100%), sulphonamides (80–100%), fluoroquinolones (40–70%), macrolides (40–100%), lincosamides (10–45%), phenicols (40–70%), nitrofurans (0–25%), and glycopeptide (0–20%). This study demonstrated a sustained increase in resistance among the most commonly used antibiotics in Uganda over the five-year study period. It implies ongoing hospital-based monitoring and surveillance of AMR patterns are needed to inform antibiotic prescribing, and to contribute to national and global AMR profiles. It also suggests continued emphasis on infection prevention and control practices (IPC), including antibiotic stewardship. Ultimately, laboratory capacity for timely bacteriological culture and sensitivity testing will provide a rational choice of antibiotics for HAI.

## 1. Introduction

Healthcare Associated Infections (HAIs) are a global health challenge for patient safety, and are a major driver of antimicrobial resistance (AMR) [1], particularly in lower- and middle-income countries (LMICs) [2]. Studies in Ethiopia and Nigeria have reported HAI cumulative incidences in surgical wards of between 5.7% and 45.8% [3]. Another study of 410 patients at a large hospital in northern Uganda documented an overall HAI prevalence of 28% [4]. Prevalence of HAI was higher (47%) among surgical patients, and wound infection was three times higher among patients on emergency than elective surgery [4]. Furthermore, a study involving 83 patients with postoperative clinical surgical site infections from June to August 2015, in Mbarara regional referral hospital, southwestern Uganda, documented that Gram-negative bacteria (GNB) were predominant (65.59%), of which *Klebsiella* species were 29.03% [5]. Additionally, 86% of the aerobic bacteria isolated were multidrug resistant (MDR) [5]. All the isolates, with exception of *Enterococci* species, were resistant to ampicillin, while GNB showed high resistance to ceftriaxone, sulfamethoxazole/trimethoprim, and gentamicin [5]. Appropriate empirical treatment of bacterial infections contracted in hospital necessitates an understanding of local AMR trends, which are only attainable through regular monitoring and surveillance.

AMR is a global health and development threat which requires urgent multi-sectoral action in order to achieve the United Nations Sustainable Development Goals (SDG), including improving global public health (SDG-3) [6]. The World Health Organization (WHO) declared AMR to be one of the top three global public health threats facing humanity, and has indicated that the misuse and overuse of antimicrobials is a principal driver in the development of drug-resistant pathogens [6]. Knowledge of local and regional AMR profiles is important for clinical decision making to improve antimicrobial use, and this can only be achieved through a regular surveillance capacity which remains largely inadequate throughout East Africa [7].

Addressing HAIs relies on timely feedback of results from AMR surveillance, and is strongly recommended by WHO as part of the core components of effective national infection prevention and control (IPC) programs [8]. Effective IPC is good for both HAIs and AMR prevention. HAIs contribute significantly to morbidity and mortality, lengthened hospital stay for infected patients, and, as a result, increase costs associated with healthcare [9,10,11]. Acquisition of HAIs depends on several factors, including patient demographics, geographical location, and post-operative hospitalisation [9,10,11,12]. They are also a consequence of the failure of antibiotics to treat infections due to AMR. In LMICs, hospital laboratories often are unable to provide culture and sensitivity testing and timely reporting of the results, so clinicians must choose antibiotics empirically. The choice for empirical antimicrobial management of HAI depends on up-to-date knowledge of local AMR patterns for the antibiotics available in that setting [9,10,11].

Since 2018, the Uganda AMR National Action Plan (NAP) has been implemented to prevent, slow down, and control the spread of antibiotic resistant organisms [13]. However, there are no regular country or hospital-wide monitoring and surveillance programs of AMR, as are recommended by the WHO [14]. Importantly, a number of AMR research and surveillance programs have been established and implemented; namely, Drivers of Resistance in Uganda and Malawi (DRUM), which investigates aspects of behaviour that are most important in spreading antibiotic resistance by surveying human behaviour in relation to antibiotics, water, sanitation, hygiene, and bacterial behaviour in response to these stimuli [15], as well as the UK’s Fleming Fund in AMR surveillance, which aims to increase the quantity and quality of data available to enable researchers to better understand the scale and scope of AMR in Uganda [16]. In 2020, Uganda had an estimated population of 45.7 million, according to the United Nations [17]. The country’s health spending per capita in 2019 was USD 44.00 [18] and Gross Domestic Product per capita was USD 956.90 [19]. The three most common causes of death in Uganda were: (i) communicable, maternal, neonatal, and nutritional diseases, (ii) non-communicable diseases, and (iii) injuries [20]. Given the prominence in Uganda of the Mulago National Referral Hospital (MNRH), there are no AMR trend analyses performed to date, so we studied AMR patterns in the surgical wards from 2014 to 2018 with a view to providing information about better choices of appropriate antibiotics in clinical situations.

Specifically, we aimed to describe common HAI bacterial pathogens, including *Klebsiella* spp., *E. coli*, *Proteus* spp., *Enterobacter* spp., *Citrobacter* spp., *Serratia marcescens*, *Providencia* spp., *Acinetobacter* spp., *Pseudomonas* spp., *Staphylococcus* spp., *Enterococcus* spp., *Streptococcus* spp., and Corynebacteria, to assess resistance patterns for specific antibiotics and describe trends in antibiotic resistance in the surgical wards of MNRH from 2014 to 2018.

## 2. Materials and Methods

### 2.1. Study Design

This was a descriptive cross-sectional study. We extracted a total of 428 culture and sensitivity results from five consecutive years (2014 to 2018) from the Laboratory Information Management System (LIMS) of Clinical Microbiology Laboratory at Makerere University College of Health Sciences, located in MNRH complex: 130 samples in 2014, 67 in 2015, 67 in 2016, 81 in 2017, and 85 in 2018.

### 2.2. Setting

#### 2.2.1. General Setting

Uganda’s public hospitals had a surgical volume of 36,670 cases in 2018, with women most commonly undergoing surgery (78.3%), with a mean age of 26.9 years [21]. Overall case distribution was 69% obstetrics/gynaecology, 24% general surgery, 4% orthopaedics, and 3% other subspecialties [21].

#### 2.2.2. Specific Setting

Laboratory analysis of samples was carried out at the Clinical Microbiology Laboratory at Makerere University College of Health Sciences, located in MNRH complex. This laboratory receives and processes clinical samples from MNRH wards. We utilized data from all bacteriologically positive samples obtained from obstetrics/gynaecology, general surgery, and orthopaedic wards of MNRH. To perform this, our study accessed the LIMS of the Clinical Microbiology Laboratory. This clinical laboratory is accredited by the College of American Pathologists. MNRH was founded in 1913 and is a teaching hospital for Makerere University College of Health Sciences. It also serves as a general hospital for the Kampala metropolitan area, whose population is 1.3 million [17]. Established hospital bed capacity is 1790, offering both public and private health services [22].

### 2.3. Variables and Data Acquisition

The following variables were extracted and included in the study: sample type, name of the surgical ward, and bacterial culture, as well as sensitivity of test results. Antibiotic classes and abbreviations can be seen in Table 1.

### 2.4. Data Collection

The study data were collected by trained research assistants from the LIMS and entered into Microsoft Excel software for Windows 10. For data validity, all collected data were crosschecked for errors.

### 2.5. Microbiological Process of Phenotypic Resistance

Our study included laboratory data on the antimicrobial susceptibility profiles of Gram-negative and Gram-positive bacterial isolates from patient samples that had originated from the obstetrics/gynaecology, general surgery, and orthopaedics wards of Mulago National Referral Hospital between 2014 and 2018 (Table 2). All samples had been bacteriologically processed according to the laboratory’s standard operating procedures. The organisms were identified using conventional biochemical tests according to the laboratory’s standard operating procedures and/or the Phoenix Automated Machine (Becton-Dickinson, Franklin Lakes, NJ, USA), according to the manufacturer’s instructions. Antimicrobial susceptibility testing was performed using the Kirby Bauer disc diffusion method, according to procedures outlined in the Clinical and Laboratory Standards Institute (CLSI), while using known control bacterial strains [23].

### 2.6. Data Analysis

The data extracted from LIMS were cleaned and analyzed using Microsoft Excel 2016 (Microsoft Corporation, Albuquerque, NM, USA) and STATA 14.0 statistical software (StataCorp, College Station, TX, USA). Descriptive analyses including frequencies, percentages, and means were performed. Cross-tabulations were used to determine the percentages of samples that were susceptible towards each antibiotic. These percentages were presented in the form of tables and figures. Resistance percentage categories were assigned for this study to determine the trends of resistance towards the antibiotics. Chi^2^ tests were used to assess the significance of differences in resistance towards the selected antibiotics for 2014, 2016, and 2018. Differences with *p*-values less than 0.05 at a 95% confidence interval were considered significant.

## 3. Results

Table 2 shows the sources and proportions of culture samples that were tested. Tracheal aspirates, pus swabs, and blood were the most common sources.

### 3.1. Bacterial Isolates from the Samples

Bacterial isolates were both Gram-positive and Gram-negative bacteria (Enterobacteriaceae and non-Enterobacteriaceae). The majority of the Enterobacteriaceae were *Klebsiella* spp. (21.7%), followed by *E. coli* (15.9%), while the majority of the non-Enterobacteriaceae were *Acinetobacter* spp. (17.5%). Gram-positives were mostly *Staphylococcus* spp. (12.4%), followed by *Enterococcus* spp. (6.1%) (Table 3).

### 3.2. Antimicrobial Susceptibility Profiles of Bacterial Isolates during 2014, 2016, and 2018

**Figure 1:**
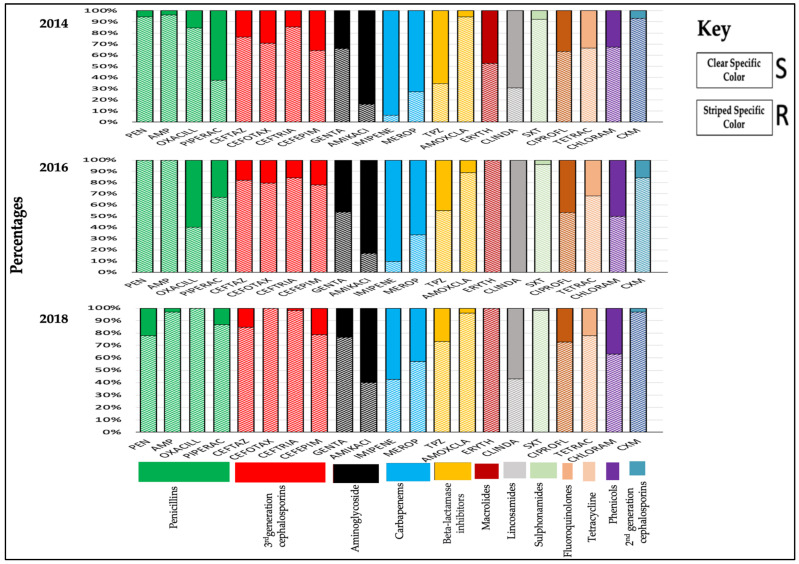
Antimicrobial susceptibility profiles of bacterial isolates from surgical wards of Mulago National Referral Hospital, Uganda, from 2014, 2016, and 2018.

Figure 1 shows the sensitivities and resistance patterns of antibiotics from 2014, 2016, and 2018. There were minor fluctuations in antibiotic resistance year by year, but, in general, certain classes of antibiotics tended to demonstrate more resistance than others.

### 3.3. Antimicrobial Resistance Trends over the Years

**Figure 2:**
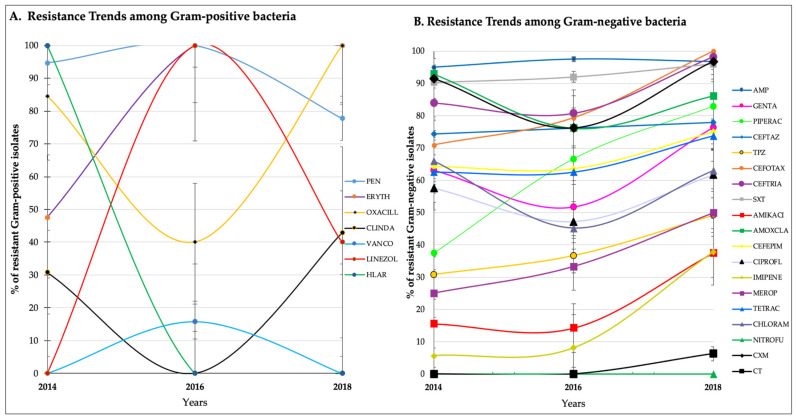
(**A**) Antimicrobial resistance trends among Gram-positive bacterial isolates for 2014, 2016, and 2018; (**B**) Antimicrobial resistance trends among Gram-negative bacterial isolates of samples collected from the surgical wards of MNRH, Uganda, for 2014, 2016, and 2018.

Among Gram-positive isolates, there were fluctuations in resistance, especially in 2016, but the trend was for an overall increase from 2014 to 2018 (Figure 2A) and (Table 4). Similarly, there was a clear trend for increased resistance among Gram-negative bacteria for virtually all antibiotics (Figure 2B) and (Table 5).

## 4. Discussion

This was the first study in Uganda to document antimicrobial resistance trends over five years in a tertiary hospital’s surgical ward. It showed a high level of resistance to many commonly prescribed antibiotics and a trend of increasing resistance to all antibiotics over five years.

This is very important because, in the absence of easily accessible laboratory-based bacteriological culture and sensitivity testing, clinicians will continue to prescribe antibiotics empirically. Knowing which classes of antibiotics have high resistance patterns becomes crucial for choosing antibiotics that are effective in treating infections. This study reinforces the practice that regular surveillance of antibiotic resistance is required to provide timely resistance pattern information to clinicians. It also underscores efforts to reduce AMR by adhering to good infection prevention and control practices.

Our findings are in agreement with a cross-sectional study from 2011 in MNRH examining bacterial isolates from surgical wards, which reported a 100% resistance to ampicillin, piperacillin, and tetracycline, and a 3.9% resistance to imipenem [24]. In other African studies, hospital-wide HAI prevalence varied between 2.5% and 14.8% in Algeria, Burkina Faso, Senegal, and the United Republic of Tanzania [3]. A study in Ethiopia from March to July 2015 observed that the prevalence of HAI was high in teaching hospitals, with surgical site infections and pneumonia being the most common [25]. This study is relevant since MNRH is the teaching hospital of Makerere University.

Previous studies on AMR analysis at MNRH [26,27,28,29] did not perform AMR trend analysis in the surgical wards. However, in Rwanda, which borders Uganda to the south west, a five-year antimicrobial susceptibility trend study among bacterial isolates from a tertiary health-care facility in Kigali, from 1 January 2009 to 31 December 2013, found high rates of resistance by Gram-negative bacteria to cephalosporins, and rising rates of resistance to last-option drugs such as imipenem and colistin [30]. Furthermore, in this study, colistin resistance was 18.5% and 3% against *Acinetobacter* spp. and *Pseudomonas* spp., respectively, whereas imipenem resistance was 54.8% and 15.7%, respectively, for the same organisms [30]. It may be important to note that, while Gram-negative bacteria are more sensitive to carbapenems compared to other beta-lactams [31], over the years, carbapenem resistance has increased. Therefore, even if this remains the choice of antibiotic for drug resistant bacteria, increasing resistance to this antibiotic poses a threat to the management of multiple drug resistant (MDR) organisms.

Our study has several operational implications. First, the rise and spread of antibiotic-resistant bacteria in hospital settings continue to reduce antibiotic options, particularly for infections involving antibiotic-resistant Gram-positive or Gram-negative bacteria. This has resulted in the increased use of last-option antibiotics, often reserved for life-threatening infections, a practice that may accelerate the development of resistance. Second, the growing trend of resistance should be a trigger to strengthen antibiotic stewardship and maximize infection prevention and control (IPC) practices to safeguard the remaining effective antibiotics. The need for local antibiotics stewardship programs cannot be overemphasized. Third, it points towards increasing laboratory capacity to perform bacteriological culture and sensitivity in a timely manner, so that antibiotics can be chosen based on evidence and not just clinical intuition. Finally, capacity needs to be built for routine AMR trend analysis in clinical microbiology laboratories to support the Ministry of Health in creating antibiotic stewardship programs.

The strengths of this study include the following: (i) data were available over a five-year period from the surgical wards of MNRH to provide a trend analysis; (ii) the bacteriological culture results came from the Clinical Microbiology Laboratory at Makerere University College of Health Sciences, which is a College of American Pathologists (CAP) accredited established facility with good quality control practices; and (iii) the study followed Strengthening the Reporting of Observational Studies in Epidemiology (STROBE) guidelines [32].

There were also some limitations, which included the following: (i) a lack of timely culture and sensitivity information available to clinicians; (ii) a small number of surgical site specimens at the time of the study; (iii) the number of sample types included in this study was constrained by the number of samples submitted to the study laboratory during the period under consideration; therefore, there was an inherent selection bias; and (iv) there was a high proportion of tracheal aspirate samples, which are not typically surgical site infections. Lastly, our analysis did not include an intermediate susceptibility category; we specifically looked at “Resistance” or “Susceptible” categories.

## 5. Conclusions

This study showed that, disturbingly, AMR increased across all antibiotic classes over five years in Mulago National Reference Hospital. It strongly suggests that ongoing surveillance be established to provide clinicians with up-to-date antibiograms to ensure that appropriate antimicrobial choices are based on the latest evidence. It also suggests that the laboratory introduces timely culture and sensitivity testing to properly target HAI pathogens.

## Figures and Tables

**Table 1:** The antibiotic classes and their respective abbreviations.

Antibiotic Classes	Antibiotics (Abbreviation)
Penicillins	penicillin (PEN), ampicillin (AMP), piperacillin (PIPERAC), piperacillin-tazobactam (TPZ), oxacillin (OXACILL), amoxicillin-clavulanic acid (AMOXCLAV)
Third generation cephalosporins	cefotaxime (CEFOTAX), ceftriaxone (CEFTRIA), ceftazidime (CAZ)
Macrolides	erythromycin (ERYTH)
Second generation cephalosporins	cefuroxime (CXM)
Sulphonamides	trimethoprim-sulfamethoxazole (SXT)
Aminoglycoside	gentamicin (GENTA), amikacin (AMIKACI), high-level aminoglycoside (HLAR),
Tetracycline	tetracycline (TETRAC)
Phenicols	chloramphenicol (CHLORAM)
Fluoroquinolones	ciprofloxacin (CIPROFL)
Carbapenems	meropenem (MEROP), imipenem (IMIPENE)
Lincosamides	clindamycin (CLINDA)
Oxazolidinones	linezolid (LINEZOL)
Polymyxin E	colistin (CT)
Nitrofurans	nitrofurantoin (NITROFU)
Glycopeptide	vancomycin (VANCO)
Fourth-generation cephalosporin	cefepime (CEFEPIM)

**Table 2:** Culture samples collected from patients attending the surgical wards of Mulago National Referral Hospital, Uganda, from 2014 to 2018.

Sample	Frequency (n = 428)	Percentage (%)
Tracheal aspirate	156	(36.5)
Pus swab	120	(28.0)
Blood	88	(20.6)
Urine	22	(5.1)
Pus aspirate	20	(4.7)
Catheter tip	13	(3.0)
High vaginal swab	3	(0.7)
Ear swab	3	(0.7)
Wound swab	2	(0.5)
Stool	1	(0.2)

**Table 3:** Bacteria isolated from samples taken from patients attending the surgical wards of Mulago National Referral Hospital, Uganda, from 2014 to 2018.

Classification		Frequency (n = 428)	Percentage (%)
Gram-negative Enterobacteriaceae (n = 216)	*Klebsiella* spp.	93	(21.7)
	*E. coli*	68	(15.9)
	*Proteus* spp.	19	(4.4)
	*Enterobacter* spp.	18	(4.2)
	*Citrobacter* spp.	11	(2.6)
	*Serratia marcescens*	4	(0.9)
	*Providencia* spp.	3	(0.7)
Gram-negative non-Enterobacteriaceae (n = 122)	*Acinetobacter* spp.	75	(17.5)
	*Pseudomonas* spp.	47	(11.0)
Gram-positive (n = 90)	*Staphylococcus* spp.	53	(12.4)
	*Enterococcus* spp.	26	(6.1)
	*Streptococcus* spp.	9	(2.1)
	Corynebacteria	2	(0.5)

**Table 4:** Resistance of Gram-positive isolates towards antibiotics from 2014 to 2018.

Antibiotic/Years	2014 n (%)	2015 n (%)	2016 n (%)	2017 n (%)	2018 n (%)	Chi^2^ Value for Trends	*p*-Value
**Penicillin**	18 (94.7)	16 (88.9)	10 (100.0)	7 (77.8)	7 (77.8)	2.13	0.144
**Erythromycin**	10 (47.6)	13 (68.4)	21 (100.0)	8 (72.7)	9 (100.0)	9.94	0.002
**Oxacillin**	11 (84.6)	6 (40.0)	3 (60.0)	2 (40.0)	4 (100.0)	0.03	0.871
**Clindamycin**	4 (30.8)	2 (18.2)	0 (0.0)	1 (14.3)	3 (42.9)	0.01	0.938
**Linezolid**	0 (0.0)	1 (50.0)	3 (100)	0 (0.0)	2 (40.0)	0.08	0.778
**HLAR**	2 (100.0)	2 (100.0)	-	2 (50.0)	0 (0.0)	3.56	0.059

There was a statistically significant increased trend in resistance towards erythromycin from 47.6% in 2014 to 100% in 2018 (x^2^ _trends_ = 9.94, *p*-value < 0.001). The bold shows statistically significant value.

**Table 5:** Resistance of Gram-negative isolates towards antibiotics from 2014 to 2018.

Antibiotic/Year	2014 n (%)	2015 n (%)	2016 n (%)	2017 n (%)	2018 n (%)	Chi^2^ Value for Trends	*p*-Value
Ampicillin	76 (95.0)	34 (100.0)	41 (97.6)	44 (97.8)	30 (96.8)	0.27	0.603
Gentamicin	70 (63.1)	19 (61.3)	30 (51.7)	42 (57.5)	55 (76.4)	0.72	0.397
Piperacillin	3 (37.5)	7 (58.3)	6 (66.7)	16 (64.0)	39 (83.0)	9.58	0.002
Cefotaxime	58 (74.4)	9 (42.9)	32 (76.2)	30 (75.0)	39 (78.0)	1.72	0.189
Piperacillin-Tazobactam	8 (30.8)	7 (21.9)	11 (36.7)	19 (41.3)	30 (49.2)	14.83	<0.001
Cefotaxime	22 (71.0)	3 (33.3)	27 (79.4)	27 (93.1)	7 (100)	8.02	0.005
Ceftriaxone	58 (84.1)	11 (61.1)	21 (80.8)	34 (89.5)	53 (98.2)	9.95	0.008
Trimethoprim-sulfamethoxazole	74 (90.2)	35 (87.5)	23 (92.0)	39 (90.7)	49 (96.1)	1.34	0.245
Amikacin	6 (15.6)	6 (15.6)	4 (14.3)	9 (20.9)	18 (37.5)	7.01	0.008
Amoxicillin clavulanic acid	52 (92.9)	31 (93.9)	16 (76.2)	24 (80.0)	25 (86.2)	0.84	0.361
Cefepime	9 (64.3)	4 (40.0)	7 (63.6)	24 (63.2)	33 (75.0)	2.25	0.134
Ciprofloxacin	38 (57.6)	18 (42.9)	17 (47.2)	38 (58.5)	37 (61.7)	2.01	0.156
Imipenem	4 (5.7)	1 (3.3)	3 (8.1)	14 (22.6)	25 (37.9)	32.29	<0.001
Meropenem	3 (25.0)	2 (100.0)	1 (33.3)	1 (14.3)	4 (50.0)	0.36	0.547
Tetracycline	10 (62.5)	8 (47.1)	15 (62.5)	8 (44.4)	14 (73.7)	0.56	0.453
Chloramphenicol	31 (66.0)	22 (50.0)	19 (45.2)	28 (59.6)	17 (63.0)	0.03	0.863
Nitrofurantoin	0 (0.0)	0 (0.0)	-	2 (22.2)	0 (0.0)	0.84	0.358
Cefuroxime	54 (91.5)	15 (53.6)	16 (76.2)	33 (89.2)	30 (96.8)	1.87	0.171
Colistin	-	0 (0.0)	0 (0.0)	0 (0.0)	1 (6.25)	0.49	0.483

Note: * *p*-value < 0.05. There was a statistically significant increased trend in resistance of the isolates towards piperacillin (x^2^ _trends_ = 9.58, *p*-value = 0.02); TPZ (x^2^ _trends_ = 14.83, *p*-value < 0.001); cefotaxime (x^2^ _trends_ = 8.02, *p*-value = 0.005); ceftriaxone (x^2^ _trends_ = 9.95, *p*-value = 0.008); amikacin (x^2^ _trends_ = 7.01, *p*-value = 0.008); and imipenem (x^2^ _trends_ = 36.4, *p*-value < 0.001) from the year 2014 to 2018. The bold shows statistically significant value.

## Data Availability

Not applicable.

## Open Access Statement and Disclaimer

In accordance with WHO’s open-access publication policy for all work funded by WHO or authored/co-authored by WHO staff members, WHO retains the copyright of this publication through a Creative Commons Attribution IGO license (http://creativecommons.org/licenses/by/4.0/igo/legalcode; accessed on 14 April 2021) which permits unrestricted use, distribution and reproduction in any medium provided the original work is properly cited. There should be no suggestion that WHO endorses any specific organization, products or services. The views expressed in this article are those of the authors and do not necessarily reflect those of their affiliated institutions. The use of the WHO logo is not permitted. This notice should be preserved along with the article’s original URL.
